# The temporal sequence and reciprocal relationships of frailty, social isolation and loneliness in older adults across 21 years

**DOI:** 10.1093/ageing/afae215

**Published:** 2024-10-03

**Authors:** Fereshteh Mehrabi, Mary Louise Pomeroy, Thomas K M Cudjoe, Emerald Jenkins, Elsa Dent, Emiel O Hoogendijk

**Affiliations:** Department of Psychology, Concordia University, Montreal, Quebec H4B 1R6, Canada; Roger and Flo Lipitz Center to Advance Policy in Aging and Disability, Department of Health Policy and Management, Johns Hopkins Bloomberg School of Public Health, Baltimore, MD21205, USA; Center for Equity in Aging, School of Nursing, Johns Hopkins University, Baltimore, MD21205, USA; Department of Medicine, Division of Geriatric Medicine and Gerontology, Johns Hopkins School of Medicine, Baltimore, MD21205, USA; Roger and Flo Lipitz Center to Advance Policy in Aging and Disability, Department of Health Policy and Management, Johns Hopkins Bloomberg School of Public Health, Baltimore, MD21205, USA; Caring Futures Institute, College of Nursing and Allied Health, Flinders University, Adelaide, Australia; Department of Epidemiology & Data Science, Amsterdam Public Health Research Institute, Amsterdam UMC – location VU University Medical Center, Amsterdam, the Netherlands

**Keywords:** social connection, longitudinal, ageing, intercept-random cross-lagged panel model, older people

## Abstract

**Background:**

It is unclear whether social isolation and loneliness may precede frailty status or whether frailty may precipitate social isolation and loneliness. We investigated the reciprocal and temporal sequence of social isolation, loneliness, and frailty among older adults across 21 years.

**Methods:**

We used seven waves of the Longitudinal Aging Study Amsterdam from 2302 Dutch older adults (M = 72.6 years, SD = 8.6, 52.1% female) ages 55 or older. Using random intercept cross-lagged panel models, we investigated between- and within-person associations of social isolation and loneliness with frailty. Frailty was measured using the Frailty Index. Loneliness was measured using the 11-item De Jong Gierveld Loneliness Scale. Social isolation was measured using a multi-domain 6-item scale.

**Results:**

Social isolation and loneliness were weakly correlated across waves. At the between-person level, individuals with higher levels of frailty tended to have higher levels of social isolation but not loneliness. At the within-person level, the cross-lagged paths indicated that earlier frailty status predicted future social isolation and loneliness over time. However, prior social isolation was not associated with subsequent frailty except at time point 5 (T5). Loneliness at specific time points (T1, T4 and T6) predicted greater frailty at later time points (T2, T5 and T7). The results also supported reciprocal and contemporaneous relations between social isolation, loneliness and frailty.

**Conclusions:**

Social isolation and loneliness are potential outcomes of frailty. Public health policies and health practitioners should prioritise interventions targeting social connection among older adults with pre-frailty or frailty.

## Key Points

- Physical frailty is a potential antecedent of social isolation over time.
- Loneliness is a potential antecedent and an outcome of frailty over time.
- There is a reciprocal and contemporaneous relationship between social isolation, loneliness, and frailty.
- Public health interventions that promote social connection among older adults with (pre) frailty are of paramount importance

## Introduction

The World Health Organisation Commission on Social Connection recently escalated social isolation and loneliness as a global public health priority [1]. Many older adults experience persistent or intermittent social isolation and loneliness [2], which have serious impacts on the physical, mental and cognitive health of ageing populations [3, 4]. Furthermore, social isolation and loneliness may be associated with frailty, an age-related state of poor health [5, 6]. From a clinical perspective, physical frailty assesses risk in older adults who cannot cope with internal and external stressors and health-related deficits [7].

The underlying mechanism for the association between social isolation and loneliness with frailty is not fully elucidated. Theoretically, one potential mechanism is that frailty decreases resiliency and biological reserves and increases vulnerability to the stress of disease and social change [7]. Likewise, social isolation and loneliness are emotionally stressful conditions that may accelerate the ageing process via increased inflammation [8]. Social isolation and loneliness may induce inflammation by impacting individuals’ physiological responses to social and biological stressors. Similarly, increasing inflammation with ageing can be a potential cause for developing frailty [9–11]. Chronic inflammation and diseases are associated with an increase in comorbidity and mortality in later life, and thus influence frailty [9, 12]. Another possibility is that socially isolated and lonely individuals may engage in unhealthy behaviours, such as poor diets, smoking, alcohol consumption and sedentary behaviours that can lead to poor health outcomes [8, 13–15]. Likewise, these high-risk lifestyles and behaviours may substantially increase the risk of developing frailty [10, 16].

According to recent systematic reviews and meta-analyses [5, 17, 18], social isolation and loneliness are cross-sectionally associated with frailty; however, a paucity of research has examined the longitudinal relationships of social isolation and loneliness with frailty [11]. Longitudinal studies [19, 20] found that high levels of social isolation or loneliness may increase the risk of transitioning from robust to pre-frailty or frailty among older adults. More specifically, isolated older adults exhibited a fourfold increased likelihood of progressing to pre-frailty after 1 year (odd ratio [OR]: 4.58: 95% confidence interval [CI]: 2.11–9.92) [19]. Likewise, Davies *et al.* [21] found that social isolation (hazard ratio [HR]: 1∙32, 1.22 to 1.43) and loneliness (HR: 2∙62, 2.49 to 2.76) were associated with an increased risk of developing frailty over 14 years. However, Ge *et al.* [22] illustrated that loneliness, not social isolation, was associated with frailty. Scarce studies have examined the impact of frailty on social isolation or loneliness [23, 24]. However, Hoogendijk *et al.* [24] found that baseline frailty was associated with loneliness but not social isolation over three years.

The aforementioned studies focused on the unidirectional associations between social isolation, loneliness, and frailty. To the best of our knowledge, only one longitudinal study has investigated the direction of associations between social isolation and loneliness simultaneously with frailty over time. Gale *et al*. [25] found a bidirectional association between loneliness and frailty phenotype, albeit frailty predicted social isolation but not vice versa. Two additional longitudinal studies examined the reciprocal association between social isolation and frailty. Maltby *et al*. [26] reported that baseline social isolation predicted the frailty index but not the phenotype of frailty over 4 years. However, both frailty indicators were predictive of social isolation over the same period. Pan *et al.* [27] observed bidirectional relationships between social isolation and the frailty index over 17 years, highlighting a ‘cumulative disadvantage’ effect.

Existing research on the associations between social isolation and frailty has predominantly relied on cross-sectional studies, with limited exploration of reverse associations. Few longitudinal studies have yielded mixed findings [25–27], and none have explored both the reciprocal and temporal sequence of relationships among social isolation, loneliness, and frailty over an extended period. Social isolation, an objective lack of social contact, and loneliness, a subjective perception of meaningful relationships, are independently linked to poor health outcomes [10]. Clarifying the directional and distinct associations between social isolation or loneliness and frailty is crucial for informing interventions to reduce social risk factors for frailty and promote healthy ageing [10]. Therefore, the present study examined (i) the temporal sequence of the relationships between social isolation, loneliness, and frailty over time and (ii) the reciprocal and contemporaneous relationships between social isolation, loneliness and frailty.

## Methods

### Study design and participants

We used data from the Longitudinal Aging Study Amsterdam (LASA), an ongoing population-based study that includes a national sample of adults ages 55 and older in the Netherlands [28]. Briefly, LASA focuses on the physical, emotional, cognitive and social aspects of functioning in older adults. Data collection was started in 1992, and participants are followed approximately every 3 years. We used the baseline data from the main interview of the second wave (1995–1996), as LASA first collected the frailty indicators at this wave (n = 2302). We included data from seven waves spanning 21 years, from 1995 to 2016. The full cohort profile is provided in Appendix 1.

### Measures

**Frailty:** We used a validated LASA Frailty Index (LASA-FI) [29], which is grounded in the accumulation of deficits model [30] and consists of 32 items. Health deficits were scored between 0 (‘no deficits’) and 1 (‘all deficits’), including self-reported chronic diseases, health status, functional limitations, Mini-Mental State Examination scores, physical performance, memory complaints and depressive symptoms. The frailty score was calculated for each participant by dividing the sum of the health deficit scores by the total number of deficits [29]. Higher values indicate greater frailty.

**Social isolation:** An established 6-item social isolation measure was adapted to the LASA dataset based on prior work conducted in the Health and Retirement Study [15, 31] and the English Longitudinal Study of Ageing [26, 32]. Respondents received one point for each of the following items: (i) being unmarried, (ii) living alone, (iii) having less than monthly contact with children, (iv) other family members, (v) or friends outside of the household and (vi) less than monthly participation in groups, clubs, organisations, or religious services. Items were summed to create a continuous score ranging from 0 to 6 with higher values indicating greater social isolation.

**Loneliness:** Loneliness was measured using the validated De Jong Gierveld (DJG) loneliness scale [33]. The DJG is an 11-item scale with a 6-item emotional loneliness subscale and a 5-item social loneliness subscale. An example of a statement includes ‘I miss having a really close friend.’ Scores ranged from 0 (‘no loneliness’) to 11 (‘severe loneliness’), with higher scores indicating greater loneliness.

**Covariates:** Based on previous studies, we considered participants’ sociodemographic characteristics and depressive symptoms as potential confounding variables at baseline [25, 26, 34, 35]. Sociodemographic characteristics included age (‘range = 58–89, in years’), sex (‘0 = male, 1 = female’) and education level in years. Depressive symptoms were measured by the Centre for Epidemiologic Studies Depression (CES-D) Scale [36]. The CES-D is a 20-item self-report scale with scores ranging from 0 to 60. Higher scores indicate greater depressive symptoms. Participants reported their depressive symptoms in the past week using a four-point Likert scale that ranged from 0 (‘rarely or never’) to 3 (‘mostly or always’). Age, education and depressive symptoms were modelled as continuous variables.

### Statistical analyses

**Figure 1:**
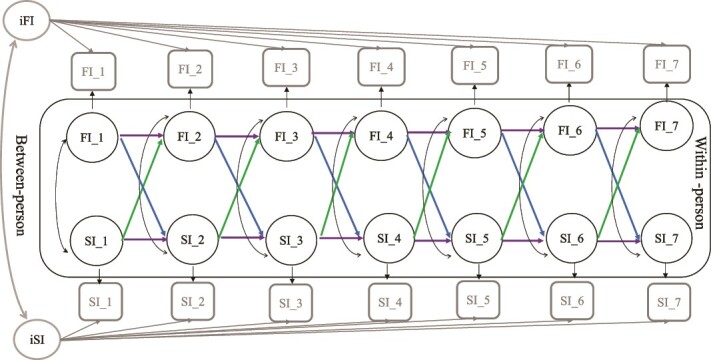
Graphical representation of the random-intercept cross-lagged panel model of social isolation and frailty index across seven waves. Notes: FI = frailty index; SI = social isolation, Squares represent observed variables. Of the observed variables, latent variables at both the between and within level were created. Circles represent latent variables. The two random intercepts (iFI and iSI) reflect the between-person variances for social isolation and frailty. The correlations between the random intercepts are represented by double arrows. The 14 latent within-person variables (FI_1–FI_7, SI_1–SI_7) reflect the within-person variances across waves 1 to 7. Factor loadings of between-person and within-person variables were constrained to 1. Double-headed arrows represent covariances. Autoregressive paths are represented by horizontal arrows, and cross-lagged paths are represented by diagonal arrows.

We conducted random intercept cross-lagged panel models (RI-CLPM) [37] to investigate the temporal dynamic relationships between social isolation, loneliness, and frailty across seven waves. The RI-CLPM includes time-invariant or ‘between-person’ effects and time-variant or ‘within-person’ effects [37]. The between-person component of the RI-CLPM includes the latent random intercept factors for social isolation, loneliness, and frailty across all time points which capture the time-invariant component of each variable (iFI and iSI in Fig. 1).

The within-person component of the RI-CLPM is represented by latent variables (black circles) based on observed variables (grey squares). All factor loadings were constrained to one and the residual measurement variances were constrained to zero.

Adjusting for the stable between-person variations, we simultaneously estimated within-person components, including autoregressive (purple horizontal arrows) and cross-lagged effects (blue and green diagonal arrows). Autoregressive effects indicate the temporal stability of a condition within an individual or within-person carryover effects in measures over time (i.e. social isolation for an individual at one time point may predict greater social isolation in the same individual at a subsequent time point). Cross-lagged effects represent how variables impact each other from one time point to the next occasion, controlling for preceding scores (i.e. social isolation for an individual at one time point may predict greater frailty in the same individual at a subsequent time point, controlling for prior frailty). We also estimated covariances between variables to examine how within-person differences in variables are correlated. All models were adjusted for time-invariant covariates (Figure 1).

RI-CLPM is criticised as assuming zero contemporaneous effects between variables while these effects may present. Hence, as suggested by Muthen and Asparouhov [38], we performed a random intercept reciprocal lagged panel model (RI-RLPM) to test the reciprocal and contemporaneous relationships between social isolation, loneliness, and frailty. The reciprocal effects assume zero cross-lagged effects (i.e. lag 1, lag 2) and test the bidirectional contemporaneous (lag 0) effects between variables. Accordingly, we performed RI-CLPM and tested autoregressive and reciprocal effects of social isolation and loneliness with frailty.

We also performed sensitivity analyses to assess the robustness of the study findings for social isolation. Given the heterogeneity in existing measures of social isolation [39], we constructed two additional versions of the social isolation measure based on previous studies. One iteration decreased the 6-item scale to a 5-item scale by omitting the item on living arrangement [21, 25, 27]. A second iteration decreased the 6-item scale to a 5-item scale by combining living arrangement and marital status into a single item [32]. We found similar trends in RI-CLPMs except for the effect of frailty at T4 on social isolation at T5, which was non-significant using the 5-item version that omits living arrangement.

We evaluated the goodness of fit based on the chi-square (χ^2^), comparative fit index (CFI), Tucker–Lewis index (TLI), the root-mean-square error of approximation (RMSEA) and the standardised root mean square residual (SRMR). CFI and TLI values of 0.95 or higher and RMSEA and SRMR values of 0.05 or lower are considered good model fit [40]. Missing data were handled with full information maximum likelihood under the assumption of missing at random. The maximum likelihood estimator with robust standard errors was used in all models. Statistical significance was set at *P* < 0.05. Data were analysed using Mplus version 8.8 [41].

## Results

### Descriptive statistics

Table 1 presents the participants’ characteristics across seven waves. The mean age of the participants was 72.6 (SD = 8.6) years, and almost half of them were women (52.1%). Participants tended to be older, female, more educated, and had more depressive symptoms over time. Consistent with prior literature [15, 42–44], social isolation and loneliness were weakly correlated within each wave (range: *r =* 0.216–0.338, *P* < 0.01) and across waves (e.g. social isolation at T2 and loneliness at T6: *r =* 0.091, *P* < 0.05; loneliness at T2 and social isolation at T3: *r =* 0.312, *P* < 0.01). The strength of the correlation between social isolation, loneliness, and frailty within the same individual also decreased over time (Appendix 2).

**Table 1:** Descriptive characteristics of the sample in seven waves

Variables	Wave 1 (n = 2302)	Wave 2 (n = 1874)	Wave 3 (n = 1474)	Wave 4 (n = 1047)	Wave 5 (n = 837)	Wave 6 (n = 704)	Wave 7 (n = 395)
Social isolation, Mean ± SD	1.92 ± 1.36	1.96 ± 1.35	1.97 ± 1.34	1.88 ± 1.3	1.97 ± 1.27	2.04 ± 1.37	2.1 ± 1.3
Loneliness, Mean ± SD	2.2 ± 2.6	2.3 ± 2.6	2.2 ± 2.6	2.2 ± 2.6	2.2 ± 2.5	2.3 ± 2.6	2.3 ± 2.5
Frailty index, Mean ± SD	0.18 ± 011	0.20 ± 0.12	0.21 ± 0.12	0.21 ± 0.12	0.23 ± 0.12	0.26 ± 0.13	0.25 ± 0.12
Age (years), Mean ± SD	72.6 ± 8.6	74.5 ± 8.3	76.2 ± 7.9	78.6 ± 7	80.5 ± 6.4	82.5 ± 5.9	84.9 ± 4.9
Sex (%)							
Male	47.9	46.5	45.9	43.7	43.4	42.9	41.5
Female	52.1	53.5	54.1	56.3	56.6	57.1	58.5
Education level (years)	9 ± 3.3	9.1 ± 3.3	9.2 ± 3.3	9.4 ± 3.2	9.6 ± 3.3	9.7 ± 3.2	10.1 ± 3.4
Depression, Mean ± SD	7.9 ± 7.8	8.6 ± 7.5	9.2 ± 7.5	8.3 ± 7.3	7.8 ± 7.2	8.3 ± 7.4	7.6 ± 6.8

Notes: SD = Standard Deviation.

### RI-CLPM results

**Figure 2:**
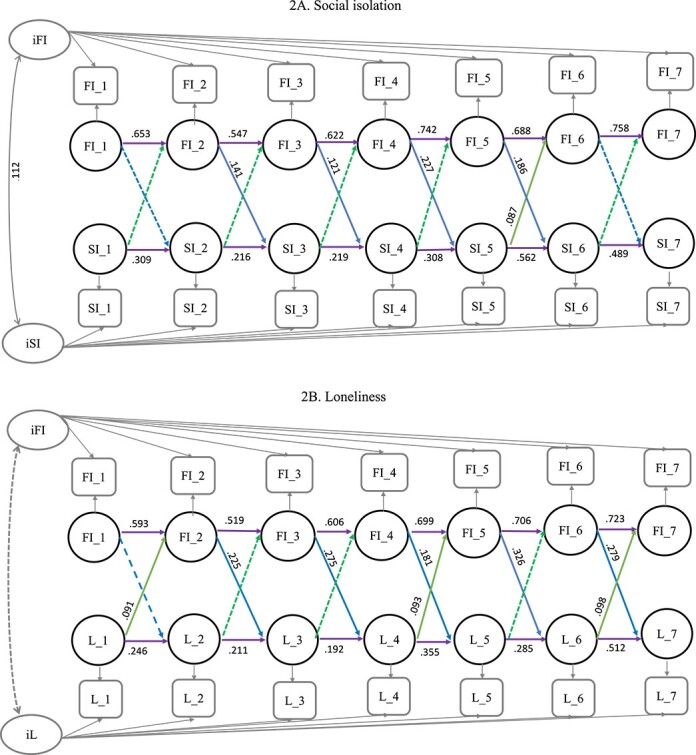
(A) A random-intercept cross-lagged panel model of social isolation and frailty index across 7 time points. Notes: FI = frailty index; SI=Social Isolation, Squares represent observed variables. Of the observed variables, latent variables at both the between and within level were created. Circles represent latent variables. Arrows represent significant paths; dashed arrows represent non-significant paths. Double-headed arrows represent correlations. Autoregressive paths are represented by horizontal arrows and cross-lagged paths are represented by diagonal arrows. Autoregressive and cross-lagged values represent standardised coefficients. T1 = Time 1 (1995–1996); T2 = Time 2 (1998–1999); T3 = Time 3 (2001–2002); T4 = Time 4 (2005–2006), T5 = Time 5 (2008–2009); T6 = Time 6 (2011–2012); T7 = Time 7 (2015–2016). For clarity, covariances between social isolation and frailty over time are omitted from the figure. (B) A random-intercept cross-lagged panel model of loneliness and frailty index across 7 time points. Notes: FI = frailty index; L = Loneliness, Squares represent observed variables. Of the observed variables, latent variables at both the between and within level were created. Circles represent latent variables. Arrows represent significant paths; dashed arrows represent non-significant paths. Double-headed arrows represent correlations. Autoregressive paths are represented by horizontal arrows and cross-lagged paths are represented by diagonal arrows. Autoregressive and cross-lagged values represent standardised coefficients. T1 = Time 1 (1995–1996); T2 = Time 2 (1998–1999); T3 = Time 3 (2001–2002); T4 = Time 4 (2005–2006), T5 = Time 5 (2008–2009); T6 = Time 6 (2011–2012); T7 = Time 7 (2015–2016). For clarity, covariances between loneliness and frailty over time are omitted from the figure.

At the between-individual level, the correlation between frailty and social isolation was statistically significant (*β* = 0.112, SE: 0.037). However, frailty was not correlated with loneliness (*β* = 0.043, SE: 0.043). Thus, individuals with higher levels of frailty tended to experience greater social isolation, but not loneliness and vice-versa (Table 2; Figure 2.).

**Table 2:** Standardised estimates of the RI-CLPM for the relationship between social isolation, loneliness and frailty across seven waves

Parameter	Estimate	SE		Estimate	SE
**Within person level**					
**Autoregressive effects**					
**Social isolation and frailty**					
FI_T1 ➝ FI_T2	0.653^***^	0.023	SI_T1 ➝ SI _T2	0.309^***^	0.047
FI_T2 ➝ FI_T3	0.547^***^	0.033	SI _T2 ➝ SI _T3	0.216^***^	0.058
FI_T3 ➝ FI_T4	0.622^***^	0.032	SI _T3 ➝ SI _T4	0.219^***^	0.053
FI_T4 ➝ FI_T5	0.742^***^	0.028	SI _T4 ➝ SI _T5	0.308^***^	0.054
FI_T5 ➝ FI_T6	0.688^***^	0.035	SI _T5 ➝ SI _T6	0.562^***^	0.049
FI_T6 ➝ FI_T7	0.758^***^	0.037	SI _T6 ➝ SI _T7	0.489^***^	0.070
**Loneliness and frailty**					
FI_T1 ➝ FI_T2	0.593^***^	0.030	L_T1 ➝ L_T2	0.246^***^	0.053
FI_T2 ➝ FI_T3	0.519^***^	0.034	L_T2 ➝ L_T3	0.211^***^	0.059
FI_T3 ➝ FI_T4	0.606^***^	0.035	L_T3 ➝ L_T4	0.192^***^	0.058
FI_T4 ➝ FI_T5	0.699^***^	0.030	L_T4 ➝ L_T5	0.355^***^	0.050
FI_T5 ➝ FI_T6	0.706 ^***^	0.037	L_T5 ➝ L_T6	0.285^***^	0.057
FI_T6 ➝ FI_T7	0.723 ^***^	0.042	L_T6 ➝ L_T7	0.512^***^	0.071
**Cross-lagged effects**					
**Social isolation and frailty**			**Frailty and social isolation**		
SI_T1 ➝ FI_T2	0.012	0.024	FI_T1 ➝ SI_T2	−0.138	0.107
SI_T2 ➝ FI_T3	−0.008	0.030	FI_T2 ➝ SI_T3	0.141^***^	0.050
SI_T3 ➝ FI_T4	−0.006	0.032	FI_T3 ➝ SI_T4	0.121^*^	0.057
SI_T4 ➝ FI_T5	0.031	0.037	FI_T4 ➝ SI_T5	0.227^***^	0.049
SI_T5 ➝ FI_T6	0.087^*^	0.037	FI_T5 ➝ SI_T6	0.186^***^	0.046
SI_T6 ➝ FI_T7	0.040	0.041	FI_T6 ➝ SI_T7	0.102	0.061
**Loneliness and frailty**			**Frailty and loneliness**		
L_T1 ➝ FI_T2	0.091^***^	0.027	FI_T1 ➝ L_T2	0.112	0.107
L_T2 ➝ FI_T3	0.030	0.031	FI_T2 ➝ L_T3	0.225^***^	0.055
L_T3 ➝ FI_T4	0.026	0.032	FI_T3 ➝ L_T4	0.275^***^	0.060
L_T4 ➝ FI_T5	0.093^***^	0.026	FI_T4 ➝ L_T5	0.181^**^	0.067
L_T5 ➝ FI_T6	0.001	0.032	FI_T5 ➝ L_T6	0.326^***^	0.065
L_T6 ➝ FI_T7	0.098^**^	0.042	FI_T6 ➝ L_T7	0.279^***^	0.077
**Covariances**					
FI_T1 with SI_T1	0.003	0.067	FI_T1 with L_T1	−0.055	0.078
FI_T2 with SI_T2	0.051	0.035	FI_T2 with L_T2	0.285^***^	0.078
FI_T3 with SI_T3	0.091^*^	0.039	FI_T3 with L_T3	0.206^***^	0.036
FI_T4 with SI_T4	0.107^*^	0.047	FI_T4 with L_T4	0.124^***^	0.038
FI_T5 with SI_T5	0.045	0.043	FI_T5 with L_T5	0.173^***^	0.045
FI_T6 with SI_T6	0.104^*^	0.046	FI_T6 with L_T6	0.172	0.046
FI_T7 with SI_T7	0.024	0.062	FI_T7 with L_T7	0.090	0.057
**Between person level**					
Intercept-FI with Intercept-SI	0.112^***^	0.037	Intercept-FI with Intercept-L	0.043	0.043

Notes: FI: Frailty Index, L: Loneliness, SI: Social Isolation, SE: standard error, RI-CLPM = random intercept cross-lagged panel model. T1 = Time 1 (1995–1996); T2 (1998–1999); T3 (2001–2002); T4 (2005–2006), T5 (2008–2009); T6 (2011–2012); T7 (2015–2016). Models were controlled for age, sex, education and depressive symptoms at baseline. Autoregressive effects capture the stability of each variable over time. Cross-lagged effects represent the impact of one variable at a previous wave on the current value of another variable. Model fit indices for RI-CLPM of social isolation and frailty: χ^2^ = 289, df = 84, *P* value ≤ 0.001, CFI = 0.984, TLI = 0.971, RMSEA = 0.033, and SRMR = 0.040. Model fit indices for RI-CLPM of loneliness and frailty: χ^2^ = 162, df = 79, *P* value ≤ 0.001, CFI = 0.993, TLI = 0.986, RMSEA = 0.021, and SRMR = 0.023. ^*^^*^^*^P < 0.001; ^*^^*^P < 0.01; ^*^P < 0.0 5.

At the within-individual level, autoregressive paths revealed high stability for frailty, social isolation and loneliness across successive occasions. This effect indicated that, for example, participants who experienced an increase in their frailty status were likely to report further increases in frailty at subsequent time points.

### Cross-lagged effects

Cross-lagged analyses showed that individuals with higher levels of frailty from Time 2 (T2) to T5 experienced greater social isolation from T3 to T6, with a particularly sizable association observed between T4 and T5 *(β* = 0.227, SE: 0.049). Likewise, frailty from T2 to T6 had significant effects on loneliness from T3 to T7. Together, these results indicated that individuals with higher levels of frailty tended to have higher levels of social isolation and loneliness over time. The exceptions were that higher baseline frailty (T1) did not predict greater social isolation and loneliness 3 years later at T2, nor did prior frailty at T6 predict subsequent social isolation at T7.

Regarding the effects of social isolation and loneliness on frailty, no significant relations occurred between social isolation and frailty over time except for the significant effect of social isolation at T5 on frailty at T6. Higher levels of loneliness at baseline (T1), T4, and T6 predicted increased frailty at T2, T5, and T7, respectively. This pattern did not emerge between loneliness at T2/T3 and T5 and frailty at T3/T4 and T6 (Table 2; Figure 2.).

Considering both effects, higher frailty at T5 predicted greater loneliness at T6 (*β* = 0.326, SE: 0.065) which subsequently led to increased frailty at T7 (*β* = 0.098, SE: 0.042). This vicious cycle between frailty and loneliness was also evident from T3 to T5. Likewise, greater frailty at T4 predicted social isolation at T5, subsequently leading to increased frailty at T6 (*β* = 0.087, SE: 0.037 (Appendix 3).

### Reciprocal effects

All autoregressive paths between loneliness and frailty as well as social isolation and frailty were significant and stable across waves. Individuals with higher levels of frailty or social isolation and loneliness at one occasion were more likely to experience increased frailty or social isolation and loneliness at subsequent occasions. Additionally, social isolation and loneliness exhibited reciprocal and contemporaneous relationships with frailty, suggesting that increases in loneliness were associated with simultaneous increases in frailty, and vice-versa (Appendixes 4–7).

## Discussion

This study investigated the temporal sequence and contemporaneous reciprocal relationships between social isolation, loneliness, and frailty across seven waves of the LASA study among a national sample of older adults in the Netherlands. The auto-regressive results showed that prior social isolation and loneliness predicted future increases in both conditions. Likewise, the effect of frailty on its own progression was consistent across waves. Individuals with frailty may have reduced resilience to external stressors as they age, with frailty being a consequence of the ageing process [6]. These findings underscore the importance of early interventions for addressing social isolation, loneliness, and frailty.

We found that older adults with frailty were more likely to experience increased social isolation and loneliness over the subsequent 18 years. Specifically, early frailty predicted social isolation in 5 out of 7 waves and loneliness in 6 out of 7 waves. Our findings align with those of Gale et al.’s [25], who demonstrated that a higher baseline frailty index was associated with an increased risk of social isolation at T4 (OR: 1.12, 95% CI:1.02, 1.23) and T5 (OR:1.16, CI: 1.06, 1.27), but not at T3. Furthermore, Gale et al.’s [25] found greater frailty at baseline was linked to higher levels of loneliness in all subsequent follow-ups [T3: OR:1.19 (CI:1.08, 1.30); T4: OR:1.20 (CI:1.10, 1.32); T5: OR:1.22 (CI:1.12, 1.34)]. There may be a cumulative effect of frailty on social isolation and loneliness as older adults with pre-frailty or frailty tend to have multiple chronic diseases, reduced physical activity or difficulties in performing activities of daily living, which may limit their social interactions, increasing the risk of social isolation [3, 17].

In examining the reverse association, we found that older adults with higher levels of social isolation did not generally experience higher levels of frailty in later life except at T5/T6. However, loneliness appeared to be a potential antecedent of physical frailty, especially in later periods (T4/T5 and T6/T7). This suggests that social isolation and loneliness are dynamic [2], leading to fluctuations in their severity, and that their interplay with frailty may vary or stabilise at certain time-points. In contrast to our results, Gale et al [25] found neither loneliness nor social isolation were associated with the future frailty index over 6 years.

Considering both directions, we found higher frailty from T3 to T5 predicted greater loneliness/social isolation from T4 toT6, which subsequently led to increased frailty from T5 toT7. Higher frailty at mid points may initiate a vicious cycle, influencing early changes in social isolation/loneliness, exacerbating health deterioration, and culminating changes in frailty. In our study, frailty had a stronger influence on social isolation and loneliness than the reverse direction. Consistent with our findings, Sha *et al*. [45] demonstrated that the impact of prior frailty (T1/T2) on subsequent loneliness (T2/T3) was greater than that of prior loneliness (T1/T2) on subsequent frailty (T2/T3). Our results differ from studies with shorter follow-up periods. Maltby *et al.* [26] found that frailty at T2 predicted social isolation at T4 and vice versa. Likewise, Pan *et al*. [27] found bidirectional associations between social isolation and physical frailty among Chinese older adults over 17 years.

However, we found reciprocal and contemporaneous relationships between social isolation, loneliness, and frailty, suggesting these conditions mutually reinforce each other in older age. Given the relatively long intervals in the LASA dataset (3 years), some non-significant cross-lagged effects might plausibly be contemporaneous, suggesting that the current status of these constructs may exert a stronger influence than their prior status.

## Strengths and limitations

The present study has several strengths. We used seven waves of the population-based LASA dataset to conduct sophisticated longitudinal models (RI-CLPM and RI-RLPM). We constructed a social isolation scale consistent with measures used in other national longitudinal datasets, allowing robust comparison across studies and contributing consistency to a field of heterogeneous measurement [39]. This study also had limitations. We controlled for depressive symptoms, some of which are reflected in the Frailty Index; however, only six out of 20 items of the CES-D were included. Previous studies on social isolation and frailty index also controlled for the CES-D [25, 26]. The attrition of participants over time may have resulted in healthier participants remaining in the dataset. We focused on the Frailty Index, though other frailty measurements can be explored in future studies.

## Implications for clinical practice, policy and research

Results from our study have important implications for clinical practice, public health policy and research. The longitudinal and bidirectional relationships between frailty, social isolation and loneliness found in the present study highlight the importance of interventions targeting all three of these constructs. Indeed, emerging research has shown that interventions to address social isolation and loneliness in community-dwelling older adults can mitigate the development and progression of frailty among older adults [46].

Our findings suggest that interventions should also target improving social connection in older adults at risk of frailty to buffer the adverse effects of social isolation or loneliness. For instance, enhancing social relationships may help to compensate for the lack of physiological reserves and age-related challenges associated with frailty (i.e. homebound status), leading to better health outcomes among older adults with increasing frailty [47, 48]. In the community setting, interventions such as social prescriptions for older adults with frailty have shown much promise. Social prescription programs involve primary care referrals to non-clinical local community services and social groups, and have been found to reduce social isolation and loneliness in older adults while enhancing resilience and wellbeing [49]. We also suggest that future multidimensional implementation trials involving older adults with (pre)frailty consider measures of social isolation and loneliness as outcome measures, particularly in low-middle income countries which have high socioeconomic inequalities in frailty [50].

## Conclusions

This longitudinal study suggests that older adults experiencing early frailty were more likely to become socially isolated or lonely in late life, though socially isolated older adults might not necessarily experience future frailty over time. Increasing frailty heightened the risk of loneliness, worsening frailty and perpetuating a vicious cycle. Efforts to reduce social isolation and loneliness and their negative impacts on health should look upstream to enhancing social connection among older adults with pre-frailty or frailty.

## Supplementary Material

aa-24-0438-File002_afae215
