# Supplementary material for: The temporal sequence and reciprocal relationships of frailty, social isolation and loneliness in older adults across 21 years
Source: Age Ageing. 2024 Oct 3;53(10):afae215. doi: 10.1093/ageing/afae215 (PMC11447375; doi:10.1093/ageing/afae215)
Supplement: aa-24-0438-File002_afae215 [file aa-24-0438-file002_afae215.docx]

**The Temporal Sequence and Reciprocal Relationships of Frailty, Social Isolation, and Loneliness in Older Adults Across 21 Years**

Table of Contents

[Appendix 1– Methods 1](#_Toc176536483)

[Appendix 1A– Study design and participants 1](#_Toc176536484)

[Appendix 1B– Flowchart of the LASA study’s completion rates and dropout reasons 3](#_Toc176536485)

[Appendix 1C– Cronbach alpha internal consistency estimates for loneliness and depressive symptoms across seven waves 4](#_Toc176536486)

[Appendix 2- Correlations between social isolation, loneliness, and frailty over time 5](#_Toc176536487)

[Appendix 3 – Random-intercept cross-lagged panel models of social isolation, loneliness and frailty across time 6](#_Toc176536488)

[Appendix 4. A random-intercept reciprocal lagged panel model of social isolation and frailty index. 7](#_Toc176536489)

[Appendix 5. A random-intercept reciprocal lagged panel model of loneliness and frailty index. 8](#_Toc176536490)

[Appendix 6– Standardised estimates of the RI-RLPM relationships between social isolation and frailty 9](#_Toc176536491)

[Appendix 7 – Standardised estimates of the RI-RLPM relationships between loneliness and frailty 10](#_Toc176536492)

# Appendix 1– Methods

## Appendix 1A– Study design and participants

The Longitudinal Aging Study Amsterdam (LASA) is an ongoing national population-based study in the Netherlands. LASA consists of a representative sample of older adults aged 55–84 from three regions in the Netherlands, covering diverse religious and urban-rural areas. Data collection was started in 1992 and participants are followed approximately every three years. Participants first joined the NESTOR study on Living Arrangements and Social Networks (LSN) in 1992 (wave A), with an oversampling of the oldest old and men, resulting in a sample of 3805 with a 60% response rate. The first LASA wave followed in 1993 with 3107 respondents (wave B), maintaining high response (85%) and cooperation rates (89%). Two additional cohorts were added in 2002–2003 (n= 1,002, wave 2B) and 2012–2013 (n=1, 023, wave 3B), with follow-up measurements integrating these new participants with the original cohort. By the eighth wave (wave I), 500 original cohort members were still participating, and data collection for the most recent wave (wave J) was completed in 2019.

LASA study focuses on the physical, emotional, cognitive, and social aspects of functioning in older adults. Trained interviewers collected data through face-to-face interviews in the participants’ homes, comprising both questionnaires and clinical tests. For the current study, we used the baseline data from the main interview of the second wave (1995-1996), as the frailty indicators were initially collected at this wave in the LASA study (n=2302). In total, we included data from the following seven waves that spanned 21 years: 1995–1996 (Time 1; “T1”), 1998/1999 (“T2”), 2001/2002 (“T3”), 2005/2006 (“T4”), 2008/2009 (“T5”), 2011/2012 (“T6”), and 2015/2016 (“T7”). Of the 2302 participants at baseline, 395 remained in the last follow-up in 2015–2016 [1-2].

**References**

1. Huisman M, Poppelaars J, van der Horst M, Beekman AT, Brug J, Van Tilburg TG, et al. Cohort profile: the longitudinal aging study Amsterdam. International journal of epidemiology. 2011;40(4):868-76.

2. Hoogendijk EO, Deeg DJ, de Breij S, Klokgieters SS, Kok AA, Stringa N, et al. The Longitudinal Aging Study Amsterdam: cohort update 2019 and additional data collections. European journal of epidemiology. 2020;35(1):61-74.

## Appendix 1B– Flowchart of the LASA study’s completion rates and dropout reasons

Wave 1

1995-1996

n=2302

Died (n=293)

Ineligible (n=28)

Refused (n=35)

No contact (n=10)

Missing data (n=62)

Wave 2

1998-1999

n=1874

(81%)

Wave 3

2001-2002

n=1474

(64%)

Wave 5

2008-2009

n=837

(36.4%)

Wave 4

2005-2006

n=1047

(46.7%)

Wave 7

2015-2016

n=395

(17.3%)

Wave 6

2011-2012

n=704

(30.5%)

Died (n= 262)

Ineligible (n=25)

Refused (n=38)

No contact (n=2)

Missing data (n=73)

Died (n=328)

Ineligible (n=15)

Refused (n=40)

No contact (n=14)

Missing data (n=30)

Died (n=138)

Ineligible (n=12)

Refused (n=22)

No contact (n=2)

Missing data (n=36)

Died (n=76)

Ineligible (n=7)

Refused (n=9)

Missing data (n=41)

Died (n=213)

Ineligible (n=21)

Refused (n=19)

No contact (n=2)

Missing data (n=54)

## Appendix 1C– Cronbach alpha internal consistency estimates for loneliness and depressive symptoms across seven waves

| Variables | Wave 1 | Wave 2 | Wave 3 | Wave 4 | Wave 5 | Wave 6 | Wave 7 |
| --- | --- | --- | --- | --- | --- | --- | --- |
| Loneliness | .87 | .90 | .92 | .83 | .88 | .55 | .90 |
| Depression | .85 | .76 | .80 | .80 | .80 | .79 | .79 |

Notes: The Cronbach’s alphas for the De Jong Gierveld (DJG) loneliness scale ranged from 0.87 to 0.90 across T1 to T7, indicating a very good level of internal reliability. Similarly, the Cronbach’s alphas for the Centre for Epidemiologic Studies Depression (CES-D) scale ranged from 0.79 to 0.85 across T1 to T7, also indicating a very good level of internal reliability.

# Appendix 2- Correlations between social isolation, loneliness, and frailty over time

|  | SI_1 | SI_2 | SI_3 | SI_4 | SI_5 | SI_6 | SI_7 | L_1 | L_2 | L_3 | L_4 | L_5 | L_6 | L_7 | FI_1 | FI_2 | FI_3 | FI_4 | FI_5 | FI_6 | FI_7 |
| --- | --- | --- | --- | --- | --- | --- | --- | --- | --- | --- | --- | --- | --- | --- | --- | --- | --- | --- | --- | --- | --- |
| SI_1 | 1 |  |  |  |  |  |  |  |  |  |  |  |  |  |  |  |  |  |  |  |  |
| SI_2 | .737^**^ | 1 |  |  |  |  |  |  |  |  |  |  |  |  |  |  |  |  |  |  |  |
| SI_3 | .671^**^ | .719^**^ | 1 |  |  |  |  |  |  |  |  |  |  |  |  |  |  |  |  |  |  |
| SI_4 | .586^**^ | .656^**^ | .727^**^ | 1 |  |  |  |  |  |  |  |  |  |  |  |  |  |  |  |  |  |
| SI_5 | .493^**^ | .536^**^ | .608^**^ | .735^**^ | 1 |  |  |  |  |  |  |  |  |  |  |  |  |  |  |  |  |
| SI_6 | .485^**^ | .512^**^ | .527^**^ | .693^**^ | .800^**^ | 1 |  |  |  |  |  |  |  |  |  |  |  |  |  |  |  |
| SI_7 | .388^**^ | .495^**^ | .541^**^ | .654^**^ | .676^**^ | .730^**^ | 1 |  |  |  |  |  |  |  |  |  |  |  |  |  |  |
| L_1 | .335^**^ | .298^**^ | .290^**^ | .223^**^ | .200^**^ | .110^*^ | .188^**^ | 1 |  |  |  |  |  |  |  |  |  |  |  |  |  |
| L_2 | .272^**^ | .318^**^ | .312^**^ | .262^**^ | .239^**^ | .171^**^ | .267^*^ | .693^**^ | 1 |  |  |  |  |  |  |  |  |  |  |  |  |
| L_3 | .230^**^ | .243^**^ | .338^**^ | .292^**^ | .241^**^ | .211^**^ | .269^**^ | .633^**^ | .686^**^ | 1 |  |  |  |  |  |  |  |  |  |  |  |
| L_4 | .189^**^ | .204^**^ | .250^**^ | .315^**^ | .299^**^ | .232^**^ | .288^**^ | .567^**^ | .608^**^ | .653^**^ | 1 |  |  |  |  |  |  |  |  |  |  |
| L_5 | .149^**^ | .159^**^ | .152^**^ | .219^**^ | .289^**^ | .245^**^ | .322^**^ | .532^**^ | .508^*^ | .583^**^ | .694^**^ | 1 |  |  |  |  |  |  |  |  |  |
| L_6 | .108^**^ | .091^*^ | .105^*^ | .155^**^ | .195^**^ | .216^**^ | .280^**^ | .554^**^ | .543^**^ | .569^**^ | .599^**^ | .677^**^ | 1 |  |  |  |  |  |  |  |  |
| L_7 | .146^**^ | .151^**^ | .135^**^ | .171^**^ | .216^**^ | .196^**^ | .303^**^ | .481^**^ | .480^**^ | .497^**^ | .582^**^ | .672^**^ | .727^**^ | 1 |  |  |  |  |  |  |  |
| FI_1 | .281^**^ | .262^**^ | .254^**^ | .220^**^ | .211^**^ | .141^**^ | .181^**^ | .335^**^ | .319^**^ | .275^**^ | .293^**^ | .248^**^ | .246^**^ | .208^**^ | 1 |  |  |  |  |  |  |
| FI_2 | .250^**^ | .274^**^ | .274^**^ | .237^**^ | .248^**^ | .201^**^ | .234^**^ | .291^**^ | .376^**^ | .319^**^ | .322^**^ | .262^**^ | .286^**^ | .210^**^ | .818^**^ | 1 |  |  |  |  |  |
| FI_3 | .223^**^ | .250^**^ | .281^**^ | .238^**^ | .258^**^ | .230^**^ | .284^**^ | .258^**^ | .299^**^ | .333^**^ | .305^**^ | .270^**^ | .284^**^ | .190^**^ | .755^**^ | .815^**^ | 1 |  |  |  |  |
| FI_4 | .165^**^ | .209^**^ | .253^**^ | .285^**^ | .305^**^ | .279^**^ | .305^**^ | .211^**^ | .255^**^ | .253^**^ | .320^**^ | .307^**^ | .339^**^ | .225^**^ | .635^**^ | .693^**^ | .783^**^ | 1 |  |  |  |
| FI_5 | .173^**^ | .201^**^ | .240^**^ | .272^**^ | .281^**^ | .290^**^ | .301^**^ | .244^**^ | .262^**^ | .277^**^ | .340^**^ | .343^**^ | .360^**^ | .269^**^ | .618^**^ | .695^**^ | .775^**^ | .818^**^ | 1 |  |  |
| FI_6 | .148^**^ | .140^**^ | .186^**^ | .254^**^ | .270^**^ | .304^**^ | .277^**^ | .268^**^ | .326^**^ | .297^**^ | .330^**^ | .325^**^ | .388^**^ | .314^**^ | .571^**^ | .616^**^ | .237^**^ | .237^**^ | .237^**^ | 1 |  |
| FI_7 | .212^**^ | .183^**^ | .220^**^ | .281^**^ | .269^**^ | .277^**^ | .282^**^ | .196^**^ | .260^**^ | .227^**^ | .300^**^ | .289^**^ | .335^**^ | .293^**^ | .498^**^ | .515^**^ | .579^**^ | .654^**^ | .695^**^ | .781^**^ | 1 |

Notes: ^**^Correlations are significant at the 0.01 level. ^*^Correlations are significant at the 0.05 level. SI: Social Isolation, L: Loneliness, FI: Frailty Index.

# Appendix 3 – Random-intercept cross-lagged panel models of social isolation, loneliness and frailty across time

Notes. This figure illustrates the combined results of the random-intercept cross-lagged panel models of loneliness, social isolation, and frailty across seven time points (Figures 2A and 2B), visually depicting the strength of the effects. FI: Frailty Index, L: Loneliness, SI: Social Isolation. T1 = Time 1 (1995–1996); T2 = Time 2 (1998–1999); T3 = Time 3 (2001–2002); T4 = Time 4 (2005–2006), T5 = Time 5 (2008–2009); T6= Time 6 (2011–2012); T7 = Time 7 (2015–2016). Arrows represent significant paths; dashed arrows represent non-significant paths.

Frailty at T2 consistently predicted both future social isolation and loneliness from T3 toT6/T7. Social isolation did not necessarily lead to future frailty, whereas loneliness predicted future frailty at certain time points. Most importantly, a vicious cycle between frailty and social isolation and loneliness emerged at mid and later time points (T3-T7), depicted with dark red and yellow arrows. Specifically, there was a continuous vicious cycle between frailty and loneliness from T3 to T7: frailty at T3 had a substantial impact on loneliness at T4, which subsequently led to frailty at T5. Likewise, frailty at T5 predicted loneliness at T6, which led to frailty at T7 (dark red arrows). Additionally, frailty at T4 predicated social isolation at T5, which led to frailty at T6 (yellow arrows).

# Appendix 4. A random-intercept reciprocal lagged panel model of social isolation and frailty index.

Notes: FI = frailty index; SI = Social Isolation, Squares represent observed variables. Of the observed variables, latent variables at both the between and within levels were created. Circles represent latent variables. Arrows represent significant paths; dashed arrows represent non-significant paths. Double-headed arrows represent covariances. Autoregressive paths are represented by horizontal arrows (purple) and reciprocal paths are represented by vertical arrows (blue and green). Autoregressive and cross-lagged values represent standardised coefficients. T1 = Time 1 (1995–1996); T2 = Time 2 (1998–1999); T3 = Time 3 (2001–2002); T4 = Time 4 (2005–2006), T5 = Time 5 (2008–2009); T6= Time 6 (2011–2012); T7 = Time 7 (2015–2016). For clarity, covariances between social isolation and frailty over time are omitted from the figure.

# Appendix 5. A random-intercept reciprocal lagged panel model of loneliness and frailty index.

Notes: FI = frailty index; L=Loneliness, Squares represent observed variables. Of the observed variables, latent variables at both the between and within levels were created. Circles represent latent variables. Arrows represent significant paths; dashed arrows represent non-significant paths. Double-headed arrows represent covariances. Autoregressive paths are represented by horizontal arrows (purple) and reciprocal paths are represented by vertical arrows (blue and green). Autoregressive and cross-lagged values represent standardised coefficients. T1 = Time 1 (1995–1996); T2 = Time 2 (1998–1999); T3 = Time 3 (2001–2002); T4 = Time 4 (2005–2006), T5 = Time 5 (2008–2009); T6= Time 6 (2011–2012); T7 = Time 7 (2015–2016). For clarity, covariances between loneliness and frailty over time are omitted from the figure.

# Appendix 6– Standardised estimates of the RI-RLPM relationships between social isolation and frailty

| **Parameter** | **Estimate** | **SE** |  | **Estimate** | **SE** |
| --- | --- | --- | --- | --- | --- |
| **Autoregressive effects** |  | |  | | |
| FI_T1 FI_T2 | 0.633^***^ | 0.024 | SI_T1 SI_T2 | 0.335^***^ | 0.041 |
| FI_T2 FI_T3 | 0.526^***^ | 0.032 | SI_T2 SI_T3 | 0.276^***^ | 0.051 |
| FI_T3 FI_T4 | 0.603^***^ | 0.032 | SI_T3 SI_T4 | 0.237^***^ | 0.051 |
| FI_T4 FI_T5 | 0.689 ^***^ | 0.035 | SI_T4 SI_T5 | 0.294^***^ | 0.049 |
| FI_T5 FI_T6 | 0.680^***^ | 0.036 | SI_T5 SI_T6 | 0.533^***^ | 0.045 |
| FI_T6 FI_T7 | 0.668 ^***^ | 0.046 | SI_T6 SI_T7 | 0.434^***^ | 0.062 |
| **Reciprocal effects** |  |  |  |  |  |
| SI_T2 FI_T2 | 0.099^**^ | 0.040 | FI_T2 SI_T2 | 0.260^***^ | 0.031 |
| SI_T3 FI_T3 | 0.093^**^ | 0.038 | FI_T3 SI_T3 | 0.278^***^ | 0.034 |
| SI_T4 FI_T4 | 0.084^**^ | 0.034 | FI_T4 SI_T4 | 0.306^***^ | 0.036 |
| SI_T5 FI_T5 | 0.090^**^ | 0.037 | FI_T5 SI_T5 | 0.288^***^ | 0.034 |
| SI_T6 FI_T6 | 0.098^**^ | 0.041 | FI_T6 SI_T6 | 0.264^***^ | 0.032 |
| SI_T7 FI_T7 | 0.095^**^ | 0.039 | FI_T7 SI_T7 | 0.272 ^***^ | 0.035 |
| **Covariances** |  |  |  |  |  |
| FI_T1 with SI_T1 | 0.084 | 0.060 |  |  |  |
| FI_T2 with SI_T2 | -0.247^***^ | 0.064 |  |  |  |
| FI_T3 with SI_T3 | -0.227^***^ | 0.068 |  |  |  |
| FI_T4 with SI_T4 | -0.225^***^ | 0.069 |  |  |  |
| FI_T5 with SI_T5 | -0.295^***^ | 0.072 |  |  |  |
| FI_T6 with SI_T6 | -0.234^***^ | 0.077 |  |  |  |
| FI_T7 with SI_T7 | -0.312^***^ | 0.085 |  |  |  |
| Intercept-FI with Intercept-SI | 0.060 | 0.040 |  |  |  |

Notes: FI: Frailty Index, SI: Social isolation, SE: standard error, RI-RLPM = random intercept reciprocal lagged panel models. T1 = Time 1 (1995–1996); T2 (1998–1999); T3 (2001–2002); T4 (2005–2006), T5 (2008–2009); T6(2011–2012); T7 = Time 7 (2015–2016). Models were controlled for age, sex, education, and depressive symptoms at baseline. Autoregressive effects capture the stability of each variable. Reciprocal effects are time-invariant. Model fit indices: χ2 = 307, df=93, p value= < .001, CFI = .983, TLI= 973, RMSEA = .032, and SRMR= .038. ****p* < .001; ***p* < .01; **p* < .05.

# Appendix 7 – Standardised estimates of the RI-RLPM relationships between loneliness and frailty

| **Parameter** | **Estimate** | **SE** |  | **Estimate** | **SE** |
| --- | --- | --- | --- | --- | --- |
| **Autoregressive effects** |  | |  | | |
| FI_T1 FI_T2 | 0.491^***^ | 0.035 | L_T1 L_T2 | 0.249^***^ | 0.042 |
| FI_T2 FI_T3 | 0.452^***^ | 0.034 | L_T2 L_T3 | 0.210^***^ | 0.047 |
| FI_T3 FI_T4 | 0.554^***^ | 0.030 | L_T3 L_T4 | 0.187^***^ | 0.046 |
| FI_T4 FI_T5 | 0.626 ^***^ | 0.033 | L_T4 L_T5 | 0.273^***^ | 0.039 |
| FI_T5 FI_T6 | 0.608^***^ | 0.036 | L_T5 L_T6 | 0.298^***^ | 0.044 |
| FI_T6 FI_T7 | 0.605 ^***^ | 0.044 | L_T6 L_T7 | 0.379^***^ | 0.050 |
| **Reciprocal effects** |  |  |  |  |  |
| L_T2 FI_T2 | 0.265^***^ | 0.045 | FI_T2 L_T2 | 0.356^***^ | 0.027 |
| L_T3 FI_T3 | 0.236^***^ | 0.040 | FI_T3 L_T3 | 0.398^***^ | 0.030 |
| L_T4 FI_T4 | 0.212^***^ | 0.036 | FI_T4 L_T4 | 0.444^***^ | 0.033 |
| L_T5 FI_T5 | 0.211^***^ | 0.037 | FI_T5 L_T5 | 0.446 ^***^ | 0.032 |
| L_T6 FI_T6 | 0.195^***^ | 0.033 | FI_T6 L_T6 | 0.483^***^ | 0.037 |
| L_T7 FI_T7 | 0.199^***^ | 0.034 | FI_T7 L_T7 | 0.474 ^***^ | 0.038 |
| **Covariances** |  |  |  |  |  |
| FI_T1 with L_T1 | -0.058 | 0.071 |  |  |  |
| FI_T2 with L_T2 | -0.333^***^ | 0.069 |  |  |  |
| FI_T3 with L_T3 | -0.399^***^ | 0.066 |  |  |  |
| FI_T4 with L_T4 | -0.461^***^ | 0.063 |  |  |  |
| FI_T5 with L_T5 | -0.433^***^ | 0.071 |  |  |  |
| FI_T6 with L_T6 | -0.427^***^ | 0.070 |  |  |  |
| FI_T7 with L_T7 | -0.511^***^ | 0.066 |  |  |  |
| Intercept-FI with Intercept-L | 0.036 | 0.039 |  |  |  |

Notes: FI: Frailty Index, L: Loneliness, SE: standard error, RI-RLPM = random intercept reciprocal lagged panel models. T1 = Time 1 (1995–1996); T2 (1998–1999); T3 (2001–2002); T4 (2005–2006), T5 (2008–2009); T6(2011–2012); T7 = Time 7 (2015–2016). Models were controlled for age, sex, education, and depressive symptoms at baseline. Autoregressive effects capture the stability of each variable. Reciprocal effects are time-invariant. Model fit indices: χ2 = 187, df=97, p value= < .001, CFI = .992, TLI= .988, RMSEA = .020, and SRMR= .031. ****p* < .001; ***p* < .01; **p* < .05.
